# Spontaneous resolution of long-standing choroidal effusion after glaucoma drainage implant surgery without significant visual deterioration : a case report

**DOI:** 10.1186/s12886-023-03213-8

**Published:** 2023-11-16

**Authors:** Mi Sun Sung, Jong Hoon Lee, Yong-Sok Ji, Sang Woo Park

**Affiliations:** Department of Ophthalmology, Chonnam National University Medical School and Hospital, 42 Jebong-ro, Dong-gu, 61469 Gwangju, South Korea

**Keywords:** Primary open-angle glaucoma, Choroidal effusion, glaucoma drainage implant Surgery

## Abstract

**Background:**

Choroidal effusion is a common complication of glaucoma surgery. Although most cases of choroidal effusions resolve spontaneously with observation or medical management alone as intraocular pressure normalizes, surgical drainage might be needed in severe or persistent cases. Herein, we report a case of spontaneous resolution of long-standing severe choroidal effusion after Ahmed glaucoma valve implantation.

**Case presentation:**

An 85-year-old man with uncontrolled primary open-angle glaucoma and medical history of chronic kidney disease underwent uneventful Ahmed glaucoma valve implantation. On postoperative day 8, transient hypotony occurred, and large 360° peripheral choroidal detachments developed. Although the intraocular pressure increased to normal levels on postoperative day 15, choroidal effusion did not resolve. Fundus examination over 8 months showed that the large choroidal effusion persisted despite a well-controlled intraocular pressure. Laboratory test performed at preoperatively and follow-up period revealed persistently elevated potassium and creatinine levels. On postoperative 9 months, the lesion resolved spontaneously without any surgical intervention. We found that the patient’s creatinine level was normalized, pre-existing hyperkalemia was corrected, and accordingly his general condition was improved.

**Conclusions:**

Considering the underlying medical condition may be helpful in patients with persistent choroidal effusion of an unclear etiology following glaucoma filtering surgery.

## Introduction

Choroidal effusion is characterized by abnormal accumulation of fluid in the suprachoroidal space and is a relatively common complication of glaucoma surgery [1]. It usually occurs in cases of significant postoperative hypotony. Reported rates of choroidal effusions range from 2.8 to 22.6% after trabeculectomy and from 9 to 35.1% after Ahmed glaucoma valve implantation [2–6]. With the widespread use of wide-field fundus photography, the rates from recent reports tend to be higher than those from previous studies because physicians can easily detect even small choroidal effusions in the peripheral retina [7]. The majority of choroidal effusions resolve spontaneously with observation or medical management alone, if the intraocular pressure (IOP) is normalized. Surgical drainage may be required in severe or persistent cases. Indications for surgery included a flat anterior chamber with lens-cornea apposition, persistent corneal edema with a shallow anterior chamber, combined serous retinal and choroidal detachment, and a prolonged duration of effusion. Persistent choroidal effusion is associated with a risk of complications, such as reduction in best-corrected visual acuity (BCVA) or cataract formation [6]. Herein, we report a case of spontaneous resolution of long-standing severe choroidal effusion without significant visual deterioration after Ahmed glaucoma valve implantation.

## Case report

**Fig. 1:**
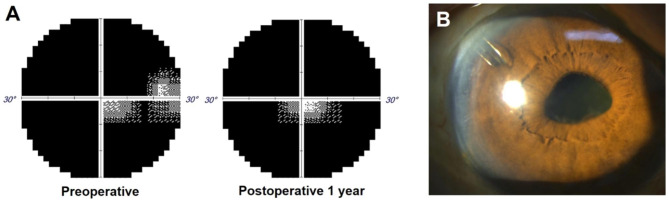
(**A**) Changes in visual field (VF) after Ahmed glaucoma valve implantation. Preoperative perimetry result shows severely constricted VF on 30 − 2 tests with small central and temporal island. At postoperative 1 year, follow-up VF test shows a well-preserved residual central island with loss of previously remaining temporal vision. (**B**) Slit-lamp anterior segment photograph showing the placement of the Ahmed glaucoma valve tube in the anterior chamber

An 85-year-old man with uncontrolled primary open-angle glaucoma, despite maximally tolerated medical therapy, was referred to our glaucoma clinic. The patient had no past history of ocular trauma, other eye disease, and intraocular surgery except for uncomplicated cataract surgery about ten years ago. He had a medical history of hypertension, type 2 diabetes mellitus, and chronic kidney disease. Routine laboratory tests performed before surgery showed electrolyte imbalance with hyperkalemia 5.7 mEq/L (normal range; 3.5–5.0 mEq/L), increased creatinine level of 1.5 mg/dL (normal range; 0.5–1.3 mg/dL), and anemia with a hemoglobin value of 10.2 g/dL. Other laboratory test results including liver function test, sodium and chloride levels, blood glucose, erythrocyte sedimentation rate (ESR), C-reactive protein (CRP), white blood cell (WBC) and platelet counts were not remarkable. Hypertension was well-controlled. Preoperative BCVA was 0.2 logarithm of the minimum angle of resolution (logMAR) in the right eye. The patient had been blinded to the left eye for the last 8 years because of uncontrolled IOP elevation and had negative light perception in the left eye. Goldmann applanation tonometry revealed that the IOP was 27 mmHg in the right eye and 40 mmHg in the left eye. Slit-lamp examination of the anterior segment revealed pseudophakia in the right eye, with no other notable abnormal findings. The anterior chamber was deep, and the angle was open to grade 4. Fundus examination of the right eye revealed optic disc pallor with large cupping and nonproliferative diabetic retinopathy. The visual field (VF) was severely constricted, with small central and temporal islands (Fig. 1A).

**Fig. 2:**
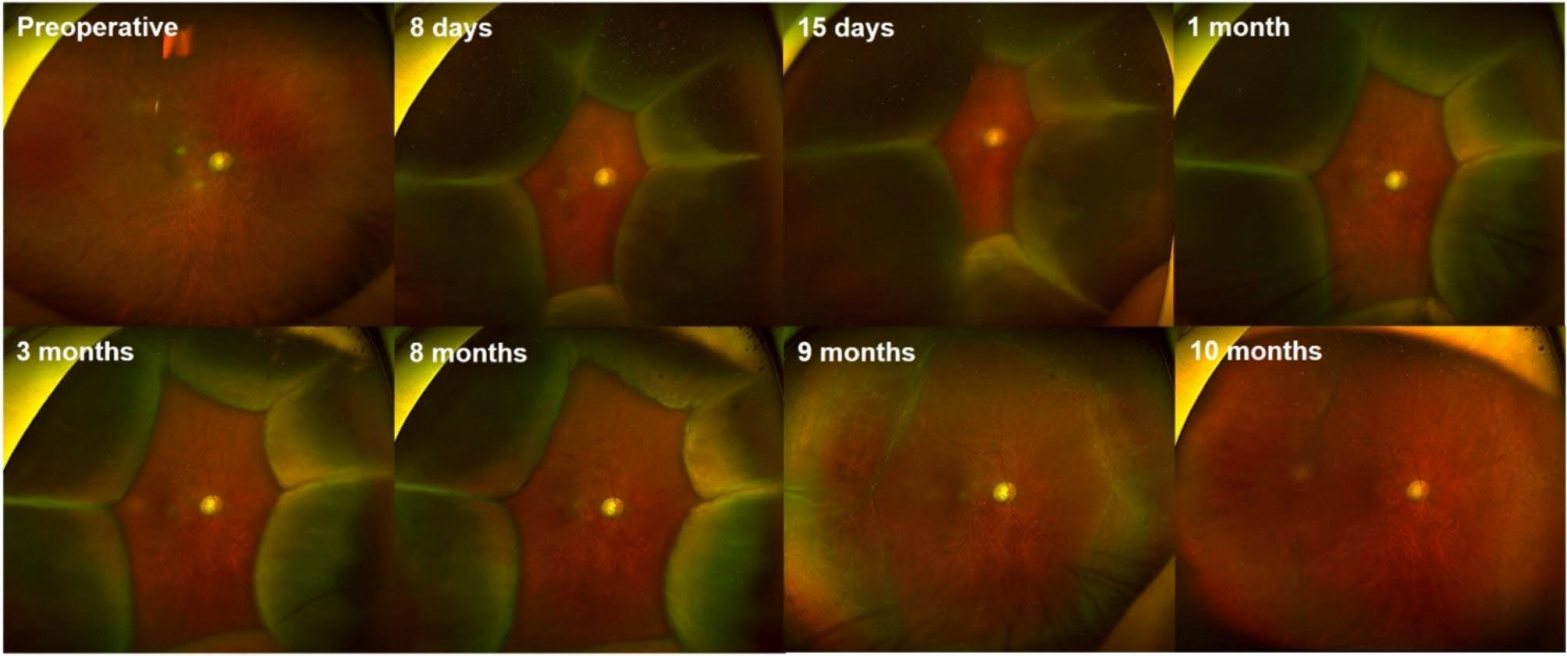
Serial ultra-wide field fundus photographs from the right eye of the patient. The image shows the development of 360° peripheral choroidal effusions with advanced glaucomatous cupping at postoperative 8 days and increased amount of choroidal effusions at postoperative 15 days. Although a subtle gradual reduction of effusions has been noted, large 360° peripheral choroidal effusions persisted until postoperative 8 months. At postoperative 9 months, marked improvement in choroidal effusion was noted, and the fundus photograph obtained at postoperative 10 months reveals a near-complete resolution of the choroidal effusion

The patient underwent an uneventful Ahmed glaucoma valve implantation and was prescribed topical antibiotic drops and corticosteroids, with instructions to discontinue prior ocular hypotensive medications. On the first operative day, the IOP decreased to 10 mmHg, with a well-maintained anterior chamber and mild inflammation. On postoperative 8 days, the anterior chamber became shallower with peripheral iridocorneal touch. The BCVA in the right eye was 1.39 logMAR with an IOP of 6 mmHg. Fundus examination revealed large peripheral choroidal detachments measuring 360°. Viscoelastic material was injected through a corneal paracentesis site to deepen the anterior chamber and increase the IOP. Topical atropine was then administered. On postoperative day 15, the IOP increased to 18 mmHg, and the anterior chamber deepened; however, the choroidal effusion did not resolve. The BCVA in the right eye was 1.22 logMAR. For the next 8 months, the IOP in the right eye was well controlled between 14 and 18 mmHg without ocular hypotensive medication, and he had no recorded episodes of anterior chamber shallowing, hypotony, or ocular inflammation; however, fundus examinations over 8 months showed that the large choroidal effusion was persistent (Fig. 2). During this period, the visual acuity slightly improved to 0.69 logMAR. However, it was still worse than the preoperative BCVA. Owing to the deterioration of the patient’s general condition, he became dependent on wheelchair and needed significant postural support during the period of choroidal effusions. Laboratory test at follow-up still revealed elevated potassium and creatinine levels and persistent anemia (potassium level of 6.0 mEq/L, creatinine level of 1.5 mg/dL, and hemoglobin value of 10.0 g/dL). There were no clinically significant changes in other laboratory parameters. He did not want any further surgical procedures, thus, additional interventions, such as drainage of the effusion, could not be performed.

On postoperative 9 months, spontaneous improvement of choroidal effusion was noted. The patient said that his systemic medical condition had markedly improved and did not need the wheelchair anymore. Follow-up laboratory tests revealed that his electrolyte imbalance was corrected and serum creatinine level was normalized (potassium level of 4.5 mEq/L and creatinine level of 1.24 mg/dL). On postoperative 10 months, the patient returned to a visual acuity of 0.2 logMAR, with a fundus examination showing near-complete resolution of the choroidal detachment. On the last visit, 1-year after the Ahmed glaucoma valve implantation, the BCVA was 0.15 logMAR, the IOP was 16 mmHg without any topical hypotensive medication, and the implant was still well functioning (Fig. 1B). A follow-up VF test revealed a well-preserved residual central island with loss of the previously remaining temporal vision (Fig. 1A).

## Discussion

The pathophysiology of choroidal effusion can be understood by considering Starling’s law between the choroidal capillaries and the interstitial space of the eyes [8]. IOP drops after glaucoma surgery are one of the main causes of choroidal effusion by increasing the hydrostatic pressure gradient and subsequently creating a pressure-driven osmotic shift of transudative fluid from choroidal capillaries into the suprachoroidal space. Early postoperative hypotony is common in clinical practice. However, not all patients with hypotony during the early postoperative period develop choroidal effusion. Several other factors have been associated with a higher risk of choroidal effusion, including older age, pseudoexfoliative glaucoma, and hypertension [2, 5, 7, 9, 10]. Recently, Ford et al. [9] demonstrated that chronic kidney disease is a significant predictor of choroidal effusions. These patient-specific preexisting factors may contribute to the structural vulnerability of the choroidal vasculature, which results in increased vascular permeability. In this case, the patient was predisposed to postoperative choroidal effusion because he was 85 years old and had a history of hypertension and chronic kidney disease.

Most postoperative choroidal effusions resolve spontaneously with conservative management alone, as the IOP normalizes. According to a report by Shin et al. [7], fluid is absorbed spontaneously approximately 12 days after effusion development, with the longest duration in uveitic glaucoma (average duration, 15.33 days). However, persistent choroidal effusion can occur even with a normal IOP [11]. Sakurai et al. recently reported a case of severe persistent chorioretinal detachment after trabeculectomy despite a normal IOP in a patient with uveitic glaucoma [11]. They suggested that chronic ocular inflammation and impaired production, outflow, or circulation of the aqueous humor might cause these complications. Similarly, our patient presented with an abnormally long-standing severe choroidal effusion for approximately 8 months in the setting of a normal IOP. However, unlike in a previous report by Sakurai et al. [11], no definitive ocular inflammation was observed during the follow-up period in this case. Furthermore, during the period of persistent choroidal effusion, there were no changes in systemic medications.

We found that the patient’s creatinine level was normalized, pre-existing hyperkalemia was corrected, and accordingly his general condition was improved, when we first noted the beginning of resolution of choroidal effusions. According to the previous study by Ford et al. [9], renin-angiotensin-aldosterone system (RAAS) is activated in kidney disease and critical illness. The RAAS cause increased intravascular pressure and promote shifting of fluid from intravascular space into the extravascular space. Thus, we postulated that impaired renal function and systemic medical conditions might alter the fluid dynamics in the body and play a role in this long-standing finding choroidal effusion. However, because of the lack of detailed medical data, the exact etiology of such long-standing choroidal effusion and the reason for the spontaneous resolution of the lesion in our patient remain unclear.

Surgical drainage should be considered for choroidal effusions that persist despite conservative treatment. Chronic non-resolving choroidal effusions cause failure of the pump mechanism of the retinal pigment epithelium, which may cause subsequent serous retinal detachment [12]. As the duration of choroidal effusion increases, the viscosity and amount of suprachoroidal fluid may also increase, making it more difficult for the fluid to be absorbed. However, due to the risk of complications from surgical intervention, it may be difficult to determine when to intervene. Therefore, the optimal timing of surgical intervention remains controversial. A recent report that retrospectively reviewed 605 patients who underwent glaucoma drainage implant surgery demonstrated that choroidal effusions developed in 110 eyes (18%) of the patients, and among the 110 eyes, surgical interventions such as drainage of choroidal effusion or implant tube ligation were performed in 19 eyes (17%) [5]. On average, surgical intervention was performed 49 days after the initial identification of choroidal effusion. In a study conducted by WuDunn et al. [13], the median time between the initial glaucoma surgery and choroidal drainage procedure was 47 days.

In the present case, choroidal drainage was not performed because the patient was in a poor general condition and did not want additional surgical intervention. He was followed up with conservative medical treatment alone, including the use of topical steroids and atropine. And at postoperative 9 months, spontaneous resolution of choroidal effusion without significant visual impairment was observed. However, we observed loss of the previously remaining temporal island on follow-up VF performed at postoperative 1 year. The effects of long-standing choroidal effusion on visual acuity and VF are not fully understood. Based on this case, it is possible that the chronic effusion in the suprachoroidal space contributed to the VF deterioration in the affected region.

To our knowledge, this is the first report of a case of spontaneously resolved long-standing choroidal effusion that lasted for more than 8 months following Ahmed glaucoma valve implantation, presumably due to underlying medical conditions. Although the management of long-standing choroidal effusions can be challenging, as they are rarely caused by a single factor, our case highlights the importance of considering the underlying medical conditions. Improving chronic medical conditions may be helpful in patients with persistent choroidal effusion of an unclear etiology following glaucoma filtering surgery.

## Data Availability

The datasets used and/or analyzed during the current study are available from the corresponding author on reasonable request.

## Abbreviations

IOP: Intraocular pressure
BCVA: Best-corrected visual acuity
logMAR: Logarithm of the minimum angle of resolution
VF: Visual field
RAAS: Renin-angiotensin-aldosterone system
